# The waiting game: when families decline – a brief commentary on delayed intervention in childhood obesity management

**DOI:** 10.1002/oby.24347

**Published:** 2025-07-01

**Authors:** José G. B. Derraik, Paul L. Hofman

**Affiliations:** ^1^ Department of Paediatrics: Child and Youth Health School of Medicine, Faculty of Medical and Health Sciences, University of Auckland Auckland New Zealand; ^2^ Environmental‐Occupational Health Sciences and Non‐Communicable Diseases Research Center Research Institute for Health Sciences, Chiang Mai University Chiang Mai Thailand; ^3^ Department of Women's and Children's Health Uppsala University Uppsala Sweden; ^4^ Liggins Institute, University of Auckland Auckland New Zealand

There is mounting global concern regarding childhood obesity, with 159 million children and adolescents aged 5 to 19 years classified as having obesity worldwide [1]. In this context, the study by Jørgensen et al. [2] investigated an important yet understudied question: whether the decision by caregivers not to engage in a family‐centered lifestyle intervention affects the long‐term weight trajectory in children with obesity.

It is commendable that Jørgensen et al. [2] capitalized on Denmark's comprehensive national health registry infrastructure to follow up children without additional participant burden. The authors linked routine anthropometric measurements from mandatory school health checks with socioeconomic data for 713 children, enabling their longitudinal tracking (median, 3.6 years) [2]. This approach minimized recall and selection biases when comparing body mass index (BMI) *z* score changes in children whose families declined a lifestyle intervention versus those who were never invited, with both groups exhibiting similar but modest BMI reductions over time. The study also highlighted the complex role of socioeconomic factors. For example, higher parental education was associated with greater BMI *z* score reduction in the group that declined participation, corroborating earlier Danish work linking lower parental education and higher rates of childhood overweight and obesity [3]. This suggests that educational attainment may facilitate healthier behaviors and contribute to reduced risk of childhood obesity.

Nonetheless, as the authors note, classifying children into a no‐intervention group leaves open the possibility that families differed in unmeasured ways, such as referral practices [4], that may have influenced comparability between groups. For example, if clinicians were less likely to invite children perceived as lower risk, the no‐intervention cohort could differ systematically from those whose families declined participation. Also, although the study [2] benefited from Denmark's routine health checks, more than the mandated three assessments were recorded for many participants, i.e., more than one‐half had four or more visits within a relatively short interval, suggesting a degree of additional monitoring that the authors themselves acknowledged. This could have fostered behavioral modifications independent of the formal intervention. The authors argued that these frequent contacts (primarily measurement‐focused and not structured interventions) were unlikely to affect long‐term weight trajectories in their study. However, regular interactions with primary care providers (especially in the context of active intervention) remain critical for sustainable behavioral change in pediatric obesity management [5].

Furthermore, interpreting short‐term changes (e.g., at 6 months) from longitudinal models, such as the adopted multiple linear splines, requires careful consideration, particularly with sparse data at early follow‐up points or when trajectories are inherently nonlinear. Although such models are powerful for describing overall trends, specific early estimates should be viewed within the broader longitudinal context, particularly considering the participants' young age (i.e., 5–8 years at obesity diagnosis). At this age, children largely depend on caregivers' decisions for study participation and lifestyle choices, influencing engagement pathways and intervention outcomes. This context of caregiver influence, in which parental decisions are paramount, aligns with the observation that BMI changes did not differ between the declined and the no‐intervention groups for these young children. Still, the absence of data on pubertal status requires caution when extrapolating study findings to older children and adolescents. These youth exhibit greater autonomy and undergo substantial physiological changes throughout puberty that are key determinants of body composition and metabolism. In addition, the apparent absence of socioeconomic or immigration status influences on weight trajectories is noteworthy but likely reflects Denmark's relatively strong social welfare system rather than universally applicable patterns. In health care systems with greater disparities, such factors would likely have a greater impact on weight gain.

Importantly, Jørgensen et al. provide valuable longitudinal data on an understudied population, i.e., children whose families declined obesity intervention. Considering the complex nature of childhood obesity, the observation that a “brief postponement” of intervention may not worsen weight trajectories could inform more thoughtful and sensitive approaches when engaging hesitant families. Existing evidence emphasizes the importance of family readiness (“readiness for change”) in pediatric obesity management, and perceived autonomy and reduced pressure from health care providers can improve engagement and adherence [6].

Although Jørgensen et al.'s findings are reassuring, the decision to postpone childhood obesity interventions must be considered with care. Childhood obesity strongly predicts obesity in adolescence and adulthood [7], and delayed intervention can negatively impact not just weight gain but also cardiovascular, metabolic, and mental health and quality of life. Therefore, the adverse health outcomes associated with childhood obesity are best addressed through early intervention. Although the present study did not examine specific treatment approaches, existing evidence shows that multidisciplinary family‐focused programs delivered at home or in community settings are most effective [8].

Although Jørgensen et al.'s observation that declining participation did not worsen weight gain compared with uninvited peers is reassuring, this should not reduce efforts to involve families in evidence‐based interventions. The option of brief postponement must be weighed against the potential benefits of early intervention. Rather than using these findings to justify delays as standard practice, they could help develop better approaches to increase family engagement. This means addressing participation barriers while respecting family choices and recognizing that brief delays may not lead to harmful outcomes in all cases. Ultimately, this Danish study [2] highlights the need for more research on the best timing and customization of interventions, especially regarding family readiness and socioeconomic factors.

## CONFLICT OF INTEREST STATEMENT

The authors declared no conflicts of interest.

## Data Availability

Data sharing is not applicable to this article as no new data were created or analyzed in this study.
